# P-1509. Disparities in Timely Administration of Nirsevimab at a Children’s Hospital During Its First Two Seasons

**DOI:** 10.1093/ofid/ofaf695.1693

**Published:** 2026-01-11

**Authors:** Tonya Scardina, Elaine Coldren, Jennifer Saper, Shreya Shriram, Rohan M Shah, Sameer Patel

**Affiliations:** Ann & Robert H. Lurie Children's Hospital of Chicago, Chicago, IL; Ann & Robert H. Lurie Children's Hospital of Chicago, Chicago, IL; Ann & Robert H. Lurie Children's Hospital of Chicago, Chicago, IL; Northwestern University Feinberg School of Medicine, Chicago, Illinois; Northwestern University Feinberg School of Medicine, Chicago, Illinois; Ann and Robert H. Lurie Children's Hospital, Chicago, IL

## Abstract

**Background:**

Nirsevimab is a long-acting monoclonal antibody designed to protect against severe respiratory syncytial virus (RSV) disease. Its initial rollout during the 2023–2024 respiratory viral season was hindered by a nationwide shortage, leading to delays and inconsistent access for eligible infants. In contrast, the 2024–2025 season saw adequate manufacturer supply. To optimize protection, timely administration of nirsevimab before peak community RSV activity is essential.

**Figure:**
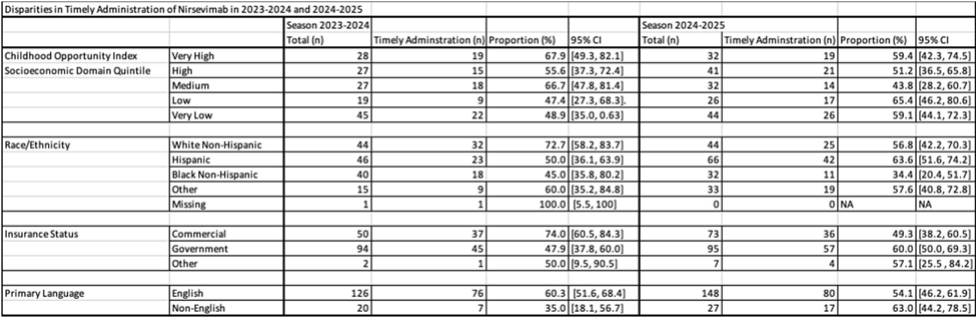

**Methods:**

For both the 2023–2024 and 2024–2025 seasons, we reviewed all nirsevimab doses administered at our children’s hospital, including inpatient and clinic settings. Included patients were infants who received nirsevimab before 8 months of age during their first RSV season, and high-risk infants eligible during their second RSV season. Electronic health record reminders and clinical care guidelines were available in both seasons. The primary outcome was timely nirsevimab administration, defined as receipt (1) before the peak of community RSV circulation (December 1 of each year) and/or (2) within 30 days of birth for infants born after December 1. We examined associations between timely administration and childhood opportunity index, insurance status, race/ethnicity, and primary language.

**Results:**

Overall, 146 patients received nirsevimab in 2023–2024 and 175 in 2024–2025. The proportion of infants receiving timely doses was similar between the two seasons: 57% (median age 2.0 months) in 2023–2024 and 55% (median age 1.6 months) in 2024–2025. In 2023–2024, timely administration differed by demographic group: white vs. black patients (72.7% vs. 45.0%, p=0.02), commercial vs. government insurance (74.0% vs. 47.9%, p< 0.01), and primary English speakers vs. other languages (60.3% vs. 35.0%, p=0.05). These disparities decreased or were no longer observed in following season.

**Conclusion:**

Among patients who received nirsevimab, disparities in timely administration were observed during the first season of availability but improved or resolved in the following year. As new medications are introduced—especially during shortages—health systems should prioritize not only overall access but also timely administration to promote immunization equity.

**Disclosures:**

Tonya Scardina, PharmD, American Society of Health System Pharmacists: Advisor/Consultant

